# Automation Advances in Stereoelectroencephalography Planning

**DOI:** 10.1016/j.nec.2020.03.005

**Published:** 2020-07

**Authors:** Vejay N. Vakharia, John S. Duncan

**Affiliations:** a Department of Clinical and Experimental Epilepsy, University College London, London, UK; b National Hospital for Neurology and Neurosurgery, Queen Square, London, UK; c Chalfont Centre for Epilepsy

**Keywords:** Stereoelectroencephalography, Computer-assisted planning, EpiNav, Epilepsy

## Abstract

The past decade has seen a significant shift in the number of centers performing intracranial electroencephalography from subdural grids and strips to stereoelectroencephalography (SEEG). Unlike grid and strip insertion or other stereotactic procedures in which the cortical surface is visualized, SEEG involves insertion of an electrode through a bolt anchored into the skull. Due to the multidisciplinary nature of SEEG trajectory planning, it often is time-consuming and iterative. Computer-assisted planning improves time taken and efficacy of SEEG trajectory planning. This article provides an overview of the considerations, controversies, and practicalities of implementing an automated computer-assisted planning solution for SEEG planning.

## Key points

• The minimum requirements for computer-assisted planning algorithms are a T1-weighted acquisition and a vascular image.
• Automated planning improves the safety and efficiency metrics of SEEG trajectories, returning feasible plans in a fraction of the time required for manual planning.
• Retrospective and prospective validation studies have returned performances that match manual expert planning when rated by external blinded experts.
• Future developments within epilepsy surgery include automated laser interstitial thermal therapy trajectory planning and machine learning algorithms to improve generalizability.

## Introduction

Stereoelectroencephalography (SEEG) is a diagnostic neurosurgical technique performed in patients with drug-refractory focal epilepsy in order to identify the seizure-onset zone when the noninvasive presurgical evaluation is nonconcordant or if surgical resection margins are not clearly defined. The planning of SEEG trajectories is a time-consuming process, and potential target structures are identified from the multidisciplinary evaluation of the clinical history, seizure semiology, scalp electroencephalography (EEG), and structural and functional imaging as well as neuropsychological and neuropsychiatric assessments. In clinical practice, once the target structures are defined (implantation strategy), surgeons follow several heuristics during SEEG trajectory planning (Box 1)

Box 1 **Stereoelectroencephalography trajectory planning heuristics**

1. Maximize distance from vasculature.
2. Avoid sulci.
3. Maximize gray matter sampling (at target and along the length of the electrode).
4. Minimize drilling angle to the skull.
5. Minimize intracerebral length.
6. Avoid critical structures.
7. Avoid other electrodes.
8. Prevent intracranial collision between electrodes.

In order to automate SEEG planning, parameter ranges for each of these factors must be identified and applied in a systematic and hierarchical manner to find the optimal solution for all electrodes in the implantation and not on an individual electrode basis. To be prospectively integrated within the SEEG pathway, the image acquisitions and preprocessing should be standardized, performed in advance of the SEEG implantation, and automated as far as possible to maximize efficiency and consistency. The selection of the anatomic structures for sampling should be available for input in a user-friendly manner and the output of the algorithm be returned within a clinically acceptable timeframe that allows the neurosurgeon and multidisciplinary team to review and modify the trajectories as needed. Finally, a method for seamlessly transferring the plan to the operating room for implantation is required. An example of one such software that has been tailor-made for this purpose is EpiNav. This is an automated stereotactic trajectory planning platform developed at University College London and has been validated for use in SEEG, laser interstitial thermal therapy, and brain biopsy planning. EpiNav is now used prospectively to plan all SEEG cases at the National Hospital for Neurology and Neurosurgery (London, United Kingdom) and has replaced manual SEEG trajectory planning since 2017. This article provides an overview of the considerations, controversies, and practicalities of implementing an automated computer-assisted planning solution for SEEG planning.

## Model generation

**Fig. 1:**
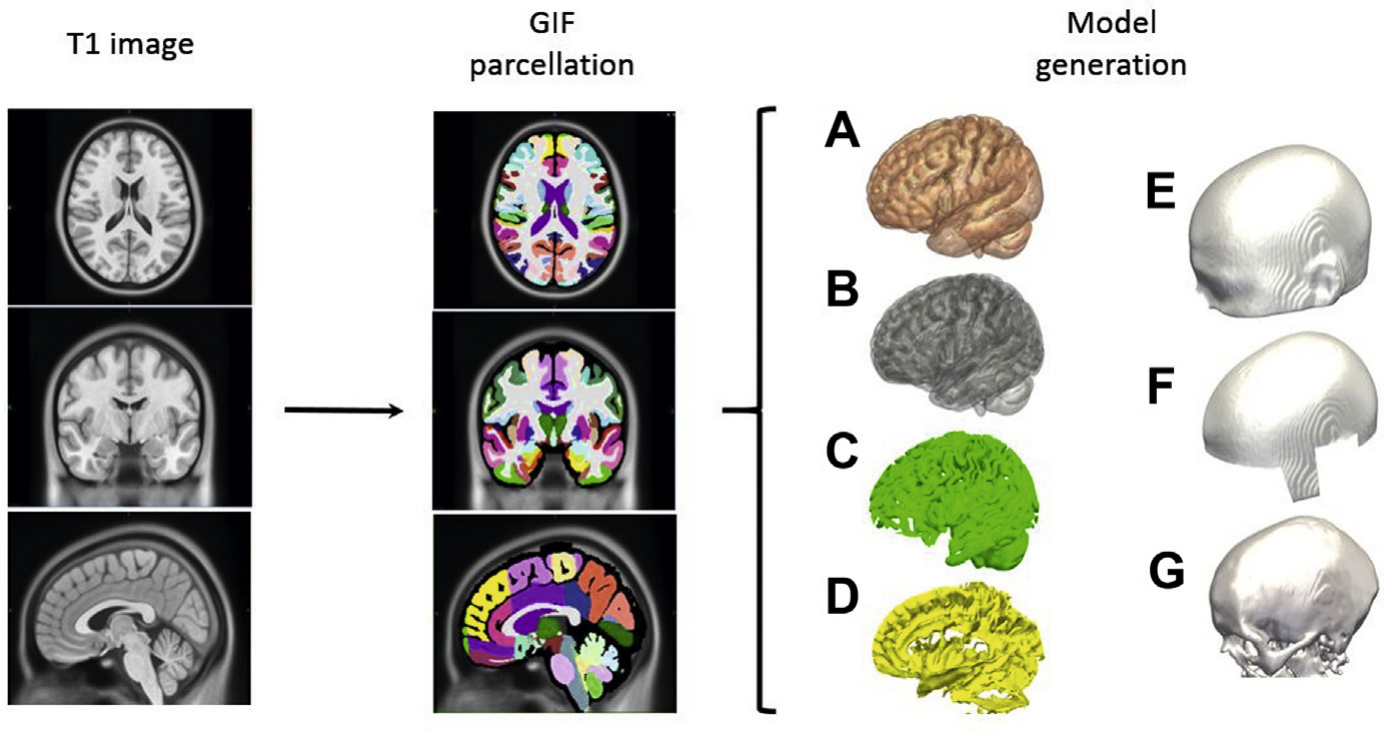
(*First column*) Input T1 image (axial (*top*), coronal (*middle*), and sagittal (*bottom*) slices shown) used to generate GIF parcellation. (*Second column*) Models generated from the GIF parcellation in an automated fashion include cortex (A), gray matter (B), gray matter–derived sulcal model (C), and CSF-derived sulcal model (D). The scalp model (E), derived directly from the T1 image through smoothing and thresholding, is modified to generate a scalp mask (F) that prevents entry through critical regions, such as the orbit, face, ear, and skull base. The skull model (G) is derived from a pseudoCT image (not shown).

The minimum requirements for performing computer-assisted planning for SEEG are a volumetric T1 magnetic resonance imaging (MRI) of the head, with and without gadolinium (Gad)-enhancement and an appropriate vascular image. The Gad-enhanced (T1+Gad) acquisition serves as the reference image and defines the planning coordinate space to which all other images are registered. The nonenhanced T1 sequence should allow differentiation between the gray and white matter, such as magnetization-prepared rapid acquisition with gradient-echo acquisitions, so that whole-brain parcellations can be derived.^1^ The whole-brain parcellation serves as an automatic means of labeling anatomic structures within the brain. The 2 whole-brain parcellations that are applied most frequently to automated SEEG planning are FreeSurfer^2^^,^^3^ and geodesic information flows (GIF),^4^ and each has its own unique advantages and disadvantages. The whole-brain parcellations allow automatic generation of 3-dimensional (D) models of the cortex, ventricular system, sulci, and gray matter (Fig. 1). The main disadvantage of a whole-brain parcellation is the time required to generate such imaging, between 2 hours and 6 hours for GIF and 10 hours and 15 hours for FreeSurfer, depending on computing power. Deep learning methods, however, have been successfully developed that could reduce this time to approximately 5 minutes,^5^^,^^6^ but clinical validation of such techniques are ongoing. The whole-brain parcellation methods were developed from structurally normal healthy brains, and, therefore, images of gross structural abnormalities, such as previous resection cavities or neonatal ischemic injuries, may be labeled incorrectly.

Trajectory distance and drilling angle metrics are measured from the external surface of the skull model. Scalp models are derived directly from the T1 MRI, whereas skull models require either pseudo–computed tomography (CT) to be generated from the T1 MRI or from a CT scan, which may have been acquired as part of previous investigations, such as positron emission tomography (PET) or single-photon emission CT (SPECT). Fig. 1 outlines the models generated from a GIF parcellation.

**Fig. 2:**
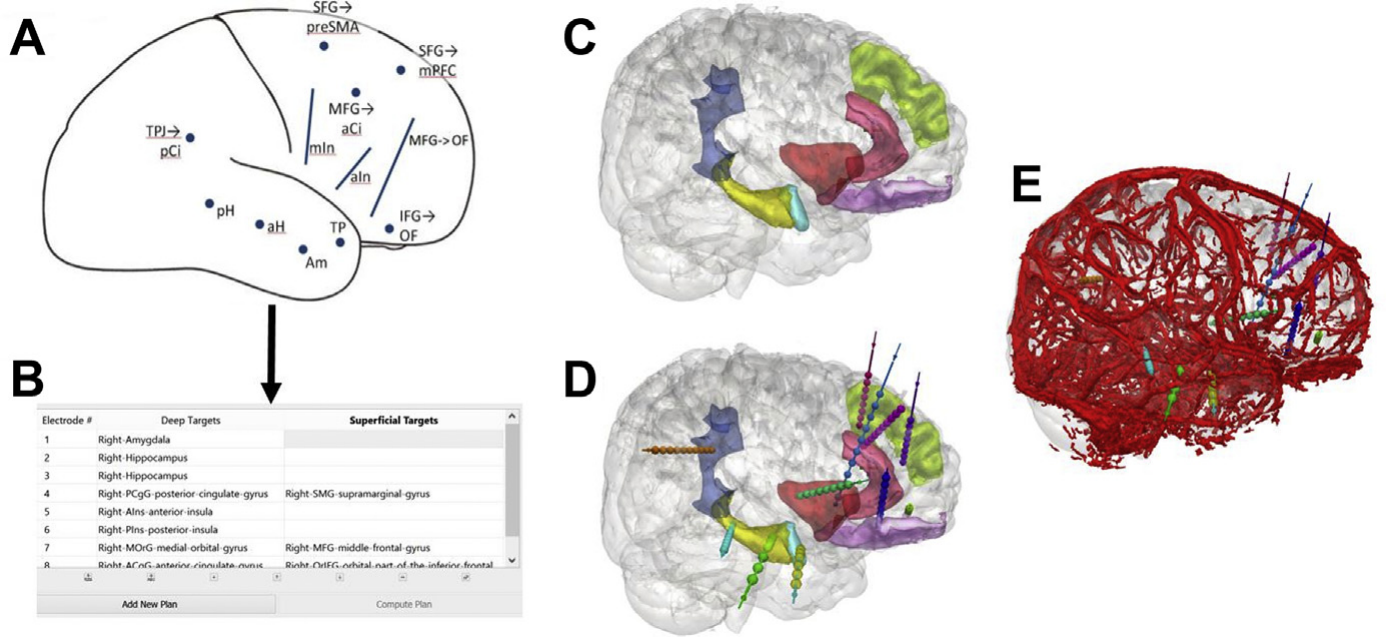
Stereoelectroencephalography (SEEG) implantation strategy (*A*) from multidisciplinary team meeting of a patient with a suspected right frontotemporal seizure-onset zone and corresponding input into (*B*) computer-assisted planning software based on the anatomically defined regions in the Geodesic information flows (GIF) parcellation. Automatic segmentation and 3-D model generation of (*C*) the anatomic structures defined as part of the implantation strategy. Corresponding automatically planned trajectories shown (*D*) without and (*E*) with the vascular model.

The user is able to select entry and target points for automated planning based on the labeled structures provided by the whole-brain parcellation. The extent to which anatomic structures are subdivided, therefore, is crucial, because this defines the precision with which the computer-assisted plans conform to the required implantation strategy. In the example outlined in Fig. 2, as part of a frontotemporal implantation strategy, the hippocampus is not subdivided into its anatomic subcomponents of head, body, and tail as part of the GIF parcellation. If 2 electrodes, therefore, are required within the hippocampus, such as 1 in the head and the other in the body, a means of ensuring this is required. One solution that has been successfully applied when 2 or more trajectories are within the same entry or target structure is to maximize their geometric spacing to ensure equal spatial sampling.

## Automated trajectory planning considerations

### Maximizing Distance from Vasculature

The greatest risk associated with SEEG trajectory planning is intracranial hemorrhage, and meta-analytic data of the published literature suggests one haemorrhage for every 29 patients implanted, with the most common type being intracerebral followed by subdural and extradural hemorrhages.^7^ Intracerebral hemorrhages most likely arise from sulcal vessels, subdural hemorrhages from cortical veins, and epidural hemorrhages from branches of the middle meningeal artery within the dura. For automated trajectory planning to avoid such hemorrhages, it is imperative that the vascular imaging modality used accurately delineate the vasculature with sufficient contrast-to-noise ratio to allow segmentation.^8^ Segmentation is the process through which the individual vessels are extracted from the source imaging. Once segmented, 3-D representations of the vascular trees can be generated and considered as structures during automated trajectory planning.^9^ This highlights the important difference between vessels that can be seen by the naked eye on the source imaging by the surgeon, compared with those that can be considered and avoided by the automated planning software.

The optimal vascular imaging modality for SEEG is still controversial.^10^, ^11^, ^12^ Digital subtraction angiography (DSA) is accepted as the gold standard method of visualizing intracranial vasculature and is undertaken by approximately half of the large volume centers currently performing SEEG.^13^ DSA, however, is an invasive procedure that involves radiation exposure and carries the risk of stroke, arterial wall dissection, and puncture site morbidity.^14^ This has led to the development and use of less invasive methods, such as CT angiography (CTA) as well as magnetic resonance (MR)-based methods.^8^^,^^15^^,^^16^ Unlike DSA, in which contrast media is injected into the internal carotid or vertebral arteries, CTA involves the injection of the contrast medium into a peripheral vein, thereby circumventing the morbidity associated with groin puncture and potential intimal vessel wall injury. The compromise, however, is that the contrast medium is diluted throughout the total circulating volume, resulting in a reduced contrast-to-noise ratio compared with DSA. MR-based methods, which include MR venography (MRV) and MR angiography (MRA), do not involve radiation exposure, and phase-contrast methods exist, which do not require the use of contrast agent administration.^17^ This is important particularly in patients with contraindications to contrast administration, such as renal dysfunction and, rarely, allergic reactions.

**Fig. 3:**
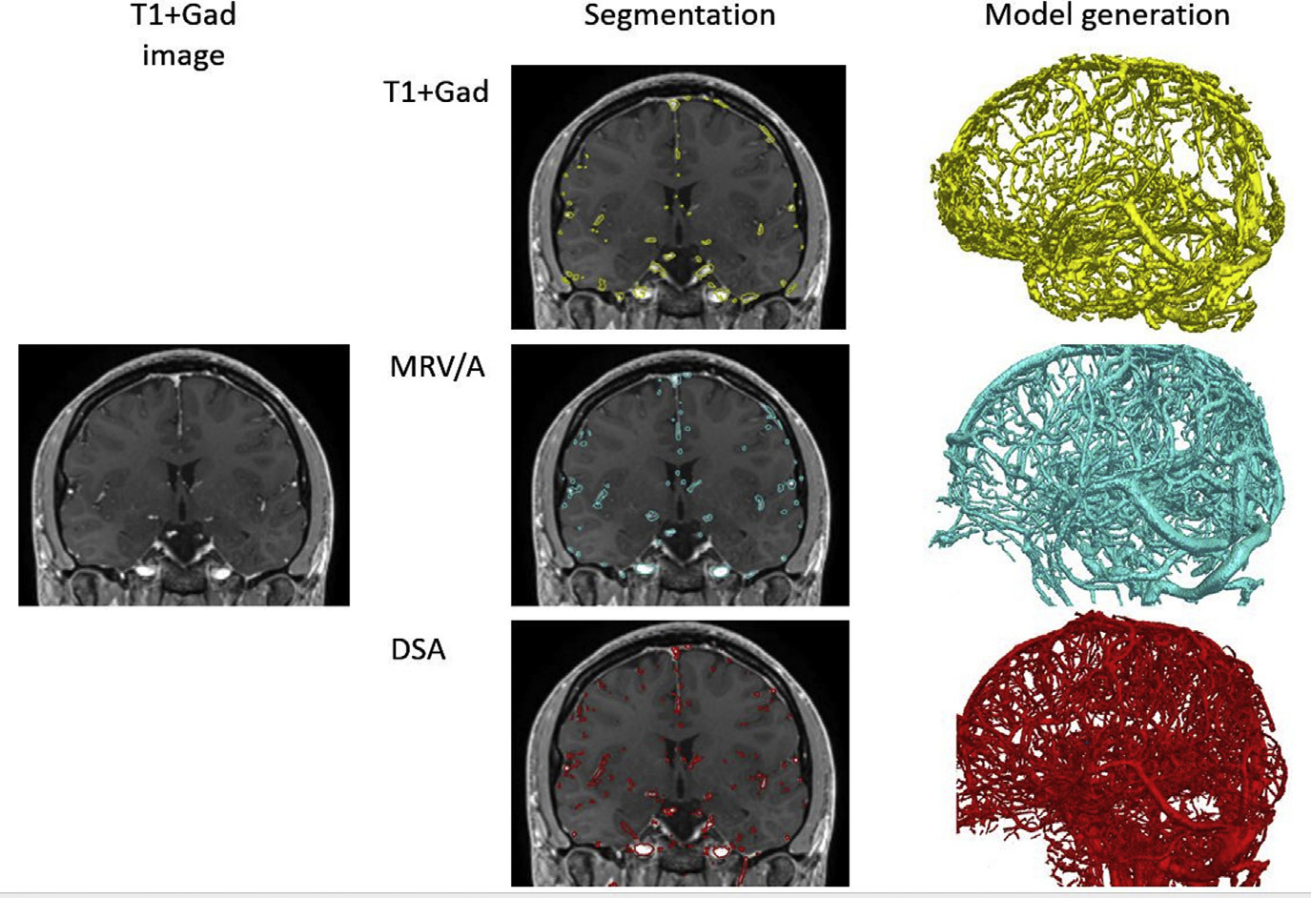
Examples of vessel segmentations from T1+Gad (*left column*), MRV/MRA (MRV/A), and DSA (*middle column*) shown in 2-D coronal planes and corresponding 3-D (*right column*) vascular model. The optimal vessel imaging technique for automated planning is based on the minimum vessel diameter considered significant by the surgeon.

Due to the low incidence of hemorrhage during SEEG, it is unlikely a comparative study between vessel imaging modalities will be performed, because prohibitively large sample sizes would be required (Fig. 3).^15^ Another important factor rarely considered in the literature is geometric distortions that are introduced through MR-based methods. Unlike with DSA/CTA, the vessels imaged through MR-based methods may not appear to be in exactly the same location due to magnetic field inhomogeneities, although distortion correction methods can be applied.^18^

Another controversial topic is the diameter of vessels that are considered to be significant for SEEG. DSA allows vessels less than 1 mm in diameter to be reliably segmented, whereas MR-based modalities are reported to be between 2 mm and 4 mm in diameter.^15^ Some studies have shown electrode-vessel conflicts with median vessel diameters less than 1.5 mm do not result in any radiological hemorrhage,^9^^,^^16^ suggesting that they may be clinically insignificant. The implication of considering clinically nonsignificant vasculature is that this may over-restrict SEEG electrode trajectory planning and unnecessarily limit the intracranial sampling.

Based on the vascular imaging method provided, the automated planning software then must calculate an optimal trajectory accounting for the location and distance from the vasculature. This is undertaken through the calculation of the minimum distance (millimeters) from the vasculature at any point along the trajectory and the risk score, which is an approximation of the size of the avascular corridor along the entire trajectory. Several different risk scores have been applied^19^, ^20^, ^21^, ^22^, ^23^ and automatically are calculated by the software by placing nodes (approximately 128) along the entire trajectory. At each of these nodal points, the closest segmented vessel is identified and the distance is measured. The closer the vessels are to the electrode the greater the risk score. The cumulative sum of the scores for all the nodal points, therefore, provides a total risk score for the entire trajectory. In addition, the user defines a safety margin, which is the minimum distance from vasculature that is clinically acceptable and is derived from the implantation accuracy achieved at that center. In general, a 3-mm to 5-mm safety margin is used.^10^ Trajectories that return nodal points less than the prespecified safety margin are discarded. The remaining trajectories then are returned based on the lowest risk score for the entire plan.^24^ The risk score is based solely on the segmentation of the vessels from the vascular imaging provided. As described previously, a poor segmentation results in fewer vessels for consideration during automated trajectory planning and falsely low apparent risk scores being returned to the surgeon.^15^ A majority of computer-assisted plans that are deemed infeasible by expert human raters are because of electrode vessel conflicts with nonsegmented vasculature.^25^, ^26^, ^27^

### Sulcal Avoidance

Anatomic dissections of the brain reveal that vessels are present within sulci, even if they cannot be visualized with the vascular imaging method used, and data from deep brain stimulation (DBS) electrode implantation suggest that crossing such pial and ependymal boundaries increases the intracranial hemorrhage rates more than 10-fold.^28^ Within the SEEG literature, however, crossing sulci improves the yield of gray matter sampling and increased hemorrhage rates have not been described.^29^ Sulcal models can be derived from the whole-brain parcellation in 1 of 2 ways. The first is to extract the intracranial cerebrospinal fluid (CSF) spaces below the crown of the gyrus. This is dependent on CSF being present within the sulci and being correctly labeled by the whole-brain parcellation. A majority of patients undergoing SEEG are young without visible CSF in the sulci, making this method less sensitive. A second method is to derive a gray matter model from the whole-brain parcellation and sequentially shrink this until it overlies the sulci (see Fig. 1). The sulcal models are considered no-go zones by some computer planning algorithms.^19^^,^^25^ No-go zones are applied in a subtly different way compared with the risk scores when considering vascular models. Here, the trajectories are permitted to run close to the sulci, in order to permit gray matter sampling, but cannot cross through them.

To date, only 2 studies have analyzed the effect of implementing sulcal models on computer-generated trajectories. In the first study,^9^ patients who had undergone SEEG implantation and also preoperative T1+Gad, MRV/MRA, and DSA imaging were retrospectively selected. The postoperative CT scan of the implanted electrodes then was overlaid on the preoperative vascular imaging. The total number of vascular conflicts between the implanted electrodes and the raw imaging then was counted. This represents the number of true conflicts. This also was compared with the number of conflicts with segmented vessels from the corresponding imaging modality. Given that computer-assisted planning algorithms can consider only segmented vasculature and the segmentation yield is dependent on the vascular imaging modality applied, this represents the apparent number of conflicts. Of 354 electrodes implanted in 33 patients, 166 electrode vessel conflicts were found on the raw DSA imaging with a median vessel size of 1.3 mm. Considering this as the number of true conflicts (ground truth), it was found that 26.5% (44/166) of the conflicts were within the gray matter–derived sulcal models, suggesting that these could have been avoided if a sulcal model was implemented during the planning. Despite there being 166 electrode vessel conflicts, however, there were no radiological hemorrhages, suggesting vessels less than 1.3 mm may be discounted during SEEG planning. In the second study,^15^ the impact of sulcal models on different vascular modalities at the planning stage was considered. Here, computer-assisted planning was performed based on providing the algorithm with DSA, MRV/MRA, and T1+Gad segmentations. In each of these cases, the plans were recomputed with and without the use of a sulcal model as no-go zones. In these cases, the use of sulcal models did not improve the risk scores significantly because the majority of the vessels within the sulci were not segmented. Overall, these studies suggest that sulcal vessels are too small to be segmented from the raw imaging and are unlikely to result in radiological or clinically significant hemorrhage. Nevertheless, further research is required before definitive conclusions can be drawn, because these studies were underpowered to detect small differences in hemorrhage rates.

### Maximize Gray Matter Sampling

**Fig. 4:**
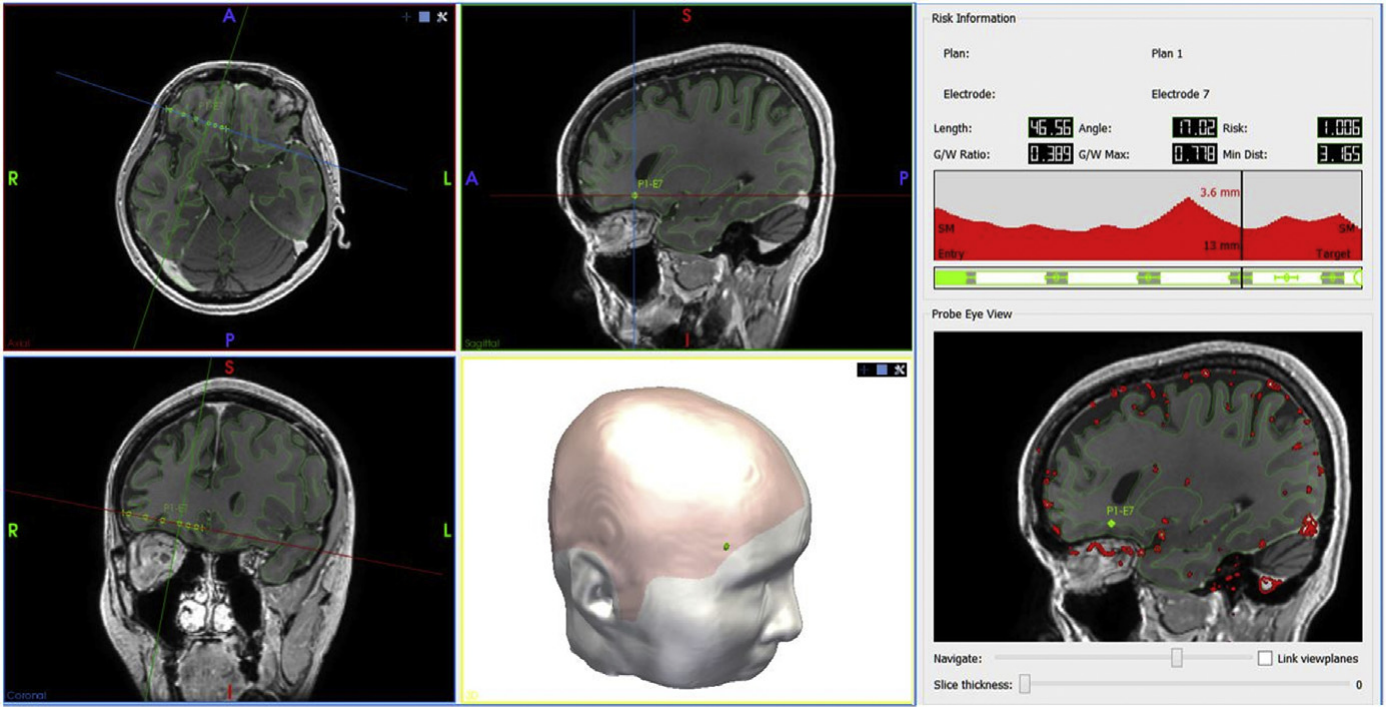
Automated right orbitofrontal electrode trajectory shown with (*top left*) axial, (*bottom left*) coronal, and (*top middle*) sagittal with planes adjusted to be orthogonal to the trajectory. Gray matter sulcal model is shown in green. Scalp model derived from (*bottom middle*) the T1 image, shown with (*pink overlay*) a scalp mask to prevent entry through the face, orbit, ear, and skull base. The right panel shows trajectory metrics, including length, drilling angle to the bone, risk score, gray-white matter sampling ratio, and minimum distance from vasculature (in millimeters). (*Top right*) Graphical display (*red*) shows distance from vasculature at varying distances from the target point, that is, at 13 mm from the target point, the closest segmented blood vessel is 3.6 mm away. (*Bottom right*) Beneath the graphical display is a pictographic representation of the electrode (*green*) and the corresponding contract position of the automatically segmented electrode that maximizes gray matter sampling. Electrode contacts that are sampling gray matter are colored gray and those in white matter are not colored. Probe eye view of the electrode along its length can be navigated using the slider to check the safety and feasibility of the trajectory.

Seizures arise within the gray matter. It, therefore, is an essential requirement of efficient SEEG sampling that the maximum possible number of electrode contacts record from gray matter. The gray matter (cortical ribbon) can be extracted directly from the whole-brain parcellation by segmentation of all cortical labels. Computer-assisted planning algorithms, therefore, calculate the proportion of the electrode that lies within the gray matter model. With prior knowledge of the electrode specification from the manufacturer, the optimal electrode can be assigned automatically to each trajectory to maximize sampling efficiency, based on the active length and contact spacing. An example of this is shown in Fig. 4, where electrodes were assigned to position contacts at gray matter interfaces and have fewer contacts in the white matter. It is necessary to have some contacts in white matter, to act as reference electrodes.

### Minimize Drilling Angle to the Skull

The skull is most reliably segmented from a plain CT scan of the head. In practice, this usually is already acquired as part of other investigations, such as PET, SPECT, and DSA imaging, and does not require additional radiation exposure. In cases where CT imaging is not available, a pseudoCT can be generated from a T1 MRI sequence. The skull model then can be can extracted from either the CT or pseudoCT image by thresholding. The skull model is used to measure the angle of electrode insertion through the skull because this determines the drilling angle. Drilling angles perpendicular to the bone are considered more accurate because they prevent the drill from sliding along the bone, a phenomenon known as skiving. As much as possible, drilling angles are limited to less than 30° to the orthogonal, although improved drill bit designs and robotic drilling platforms with increased stability likely overcome this limitation.

### Minimize Intracerebral Length

The total intracerebral length of the electrode is minimized to provide the most direct trajectory to the target structures and also minimize cerebral transgression. In practice, this also prevents the algorithm from considering contralateral entry points and returning bilateral target sampling in cases of paramedian structures, such as the cingulate cortices or the supplementary motor areas. The intracerebral length is measured from the surface of the cortical model to the target point.

### Avoidance of Critical Structures

In addition to vasculature, other critical structures that must be considered as part of automated SEEG trajectory planning include the orbit, face, skull base, posterior fossa, ears, ventricles, and basal ganglia. The skull or scalp models can be modified, either manually or by using a predefined mask, to remove the face, orbit, ears, posterior fossa, and contralateral hemisphere in cases of unilateral implantations (see Figs. 1F and 4). The mask then is used to constrain entry points for trajectory planning. The ventricular system and basal ganglia can be extracted from the whole-brain parcellation and, similar to sulcal models, designated as no-go regions. Typically, only the frontal horns, body, and occipital horns of the lateral ventricles are segmented and considered no-go zones because lateral trajectories to the hippocampus typically require crossing of the temporal horns.

### Avoid Other Electrodes

Unlike DBS implantations, where usually only 1 electrode is implanted into a single hemisphere, SEEG typically requires consideration of multiple trajectories (typically 8–14 electrodes). To prevent electrode conflict within the brain or clashing of the bolts on the scalp surface, it is common for algorithms to implement a minimum distance constraint between the trajectories. Based on user preference, a distance of 10 mm usually is employed. This adds another layer of computational complexity, because the optimal electrode for a single target point, therefore, may not be the optimal solution for the entire implantation strategy. A slightly higher risk score for a single trajectory may therefore ultimately translate to a lower overall risk score for the entire implantation. A potential advantage of this is that if an electrode trajectory is then modified or added at a later time by the user, the remaining trajectories then can be automatically recalculated and optimized without the need to laboriously manually adjust each of the other electrodes.

## Postimplantation functionality

After SEEG implantation, patients usually have CT imaging to check for any immediate postimplantation complications, such as hemorrhage, and to segment the electrodes. An MRI scan also may be performed to provide accurate data regarding the anatomic regions that have been sampled, although the artifact from the electrode contacts may be significant. From the postoperative implantation data, automated systems are available to automatically segment the individual contacts and assign them to the corresponding electrode.^30^ The purposes of this are to (1) improve understanding of which regions of the brain are being sampled, (2) allow implantation accuracy measures to be automated, (3) assess electrode bending, and (4) allow linking of the recorded EEG data to identify the relevant brain region/structure from which seizures arise.

There is a lack of uniformity in the published literature regarding how postoperative SEEG accuracy is measured and reported.^31^ Approximately half of the studies reporting post-SEEG implantation accuracy report the Euclidean distance between the planned and implemented target points, whereas the remaining half report lateral deviation. Automated systems provide a means of objectively standardizing implantation accuracy reporting.

## Clinical applications of computer-assisted planning

The first reported clinical use of computer-assisted planning software was to aid the calculation of frame-based coordinates for pediatric brain biopsy.^32^ In this study, the surgeon manually planned trajectories in 30 patients and the software was used to calculate the frame-based stereotactic coordinates of the target point. This was improved by the addition of multimodal imaging to allow coregistration of CT, MRI, and digital angiography.^33^ The next notable advance was the addition of 3-D models to trajectory planning. These early prototypes laid the foundation for computer-assisted planning but, due to the lack of computing power, were time-consuming and impractical. A resurgence in computer-assisted planning was marked by the development of computerized brain atlases,^34^^,^^35^ which were initially registered to a patient’s MRI scans and used to plan lesioning procedures for movement disorders. This was useful particularly for target structures that could not be visualized on the MRI, such as the ventral intermediate nucleus of the thalamus.

The NeuroPlanner software allowed image coregistration, integration of multiple brain atlases, 3-D model generation, manual stereotactic trajectory planning, and simulation of therapeutic lesioning and stimulation,^36^ resulting in reduced operative time, cost, and complication rates but with increased flexibility. In a path-planning algorithm that attributed cost functions to critical structures, after manual selection of a target point, the algorithm individually selected and calculated trajectories arising from entry points on the scalp model. The proximity to a critical structure, such as vasculature, incurred a cost and an overall score was attributed to each of the potential trajectories. A display of the lowest cost paths gave a risk map to aid in the selection of the optimal entry point.^37^ Systems integrating probabilistic functional targeting atlases^38^ and increasingly complex weighting systems applied to the critical structures marked further advancements in DBS planning.^22^^,^^39^, ^40^, ^41^

Table 1 provides an overview of the different planning platforms and their respective functionality as related to automated trajectory planning.

**Table 1:** Overview of the different planning platforms and their respective functionality as related to automated trajectory planning

Platform	EpiNav	SEEG assistant—3-D slicer extension	MINC Toolkit (ITK and MATLAB)	Curry
Group	UCL/King’s College London	Politecnico di Milano	Montreal Neurological Institute	Compumedics Neuroscan
Licensing	Academic use	Academic use	Academic use	Commercial
Primary use	SEEG planning	SEEG planning	SEEG planning (amygdala and hippocampus only)	Grid and strip planning
Image registration	Rigid	Rigid	Rigid	Rigid
	Affine	Affine	Affine	Affine
	Nonlinear	Nonlinear	Nonlinear	Nonlinear
Multimodal imaging	Yes	Yes	No	Yes
Vessel segmentation	Yes	Yes	Yes	No
Target structure segmentation	Yes	Yes	Yes	No
Surface/target risk map	Yes	Yes	Yes	No
Sulcal model extraction	Yes	Yes	Yes	No
Gray matter maximization	Yes	Yes	Yes	Yes
Electrode selection optimization	Yes	Yes	No	No
Single trajectory planning	Yes	Yes	Yes	No
Multitrajectory planning	Yes	Yes	Yes	No
Automated contact segmentation	Yes	No	No	Yes
Automated implantation accuracy measurement	Yes	No	No	No
Linking of intracranial EEG data to contacts	Yes	No	No	Yes
Signal processing and source reconstruction	Yes	No	No	Yes
Automated resection planning	Yes	No	No	No
Retrospective validation data	Yes—external and multicenter	Yes—internal and single center	Yes—internal and single center	N/A
Prospective validation data	Yes	No	No	N/A
Extended uses	LITT—MTLE LITT—corpus callosotomy Brain biopsy	Not specified	Not specified	Spike detection and clustering

*Abbreviations:* LITT, laser interstitial thermal therapy; MTLE, mesial temporal lobe epilepsy; N/A, not applicable; UCL, University College London.

Early studies reporting automated SEEG planning software incorporated many of the planning features that had been developed for single DBS planning. These systems additionally integrated 3-D multimodal imaging that allowed coregistration of different imaging modalities, such as fluid-attenuated inversion recovery, PET, and SPECT to aid the inference of the epileptogenic zone.^10^^,^^42^^,^^43^ The desired target points then were selected manually by the surgeon, based on the implantation strategy, and the automated planning algorithm returned the trajectory with the lowest risk based on the user-defined angle and length constraints.^44^, ^45^, ^46^ Based on a fixed target point, the trajectory risk then was represented to the user as a heat map on the scalp surface.^46^^,^^47^

Unlike DBS procedures, where the target points are stereotyped, in SEEG the target points vary significantly between electrodes and between patients. If the manually selected target points were close to a blood vessel or critical structure, the number of potential trajectory options subsequently was restricted.^26^ To prevent this restriction, some algorithms allowed the entry and target points to be roughly selected and the algorithm would expand the potential search radius by 0.5 mm.^19^ In this manner, the software was able to return the optimal solution that was most closely in keeping with the preferences of the surgeon. Using DSA-derived vasculature, this algorithm was retrospectively validated on 15 patients who underwent implantation of 199 electrodes. The automated trajectories took approximately 1 minute per electrode to be generated and when compared with the manually planned trajectories returned significantly improved distance from vasculature and insertion angles. Feasibility also was rated by 3 blinded internal neurosurgeons and the automatically generated trajectories were rated as preferable to the manual trajectories in between 50% and 73% of cases. This highlights the difference in planning practices even between neurosurgeons at the same institution. In a similar study utilizing MRV/MRA-based vasculature, the EpiNav software considered all points on the skull surface as potential entry points, thereby obviating the surgeon manually selecting a rough entry region. A total of 166 electrodes were retrospectively recreated in 18 patients and external validation from blinded neurosurgeons rated 79% of these as feasible.^26^ This method took on average 8 minutes to generate the entire implantation strategy. In these studies, however, reasons for rejecting computer-generated trajectories included conflict with nonsegmented vasculature, restrictions placed by the use of the implantation method (ie, the stereotactic frame), and potential conflict with other electrodes.

The next level of complexity introduced to automated SEEG trajectory planning was the consideration of multiple trajectories^24^^,^^27^^,^^48^ to find the global optimum solution that is the lowest overall risk for the implantation strategy as a whole, as opposed to the lowest risk for the individual trajectory. One such method utilized a serial approach in which the optimal first electrode was planned and any electrode in conflict with this subsequently was removed followed by the selection of the best next trajectory from the remaining pool.^48^ This then was repeated for all of the electrodes in the plan. Expert feasibility ratings of retrospectively reconstructed plans revealed that 30% to 40% of the automated trajectories were preferred over the manually planned trajectories. Because this method was based on the order of the trajectories considered, it could return different solutions according to the order of target regions chosen. To remove the limitation of a serial constraint and to improve computational efficiency, a dynamic programming strategy was included in the EpiNav software that was able to find combinations without limitations on the number or order of trajectories and was able to return a whole implantation strategy in less than an average of 20 seconds.^24^ Computer-generated trajectories were able to significantly reduce the risk scores and improve gray matter sampling compared with the manual trajectories.

A critical additional feature of automated trajectory planning is that the user then should be able to review the individual trajectories and modify these as required. Although manually changing the proposed entry or target point is possible, a more efficient method is to iterate through the automated trajectories in a risk-stratified manner based on the planning preference of the surgeon. In this case, a Next Entry or Next Target function allows the user to iterate through the computer-generated trajectory options until satisfied with the electrode trajectory. This involves the computation of several different putative entry and target points within the region of interest that the surgeon can evaluate. Given that these Next trajectories are derived using the multitrajectory planning algorithm, they are cognizant of other trajectories in the plan. A clinical validation study utilizing this function also was used to determine the external feasibility of the algorithm in an additional 13 consecutive patients (116 electrodes).^49^ Blinded external raters found no significant difference between feasibility ratings of the EpiNav trajectories (62%) and the manually planned trajectories (69%). The external raters in this study included neurosurgeons who performed implantations with frame-based, frameless, and robotic methods, which might explain why 30% of the manually planned trajectories were deemed infeasible despite having already been implanted in patients without complication.

To date, only a single study has reported on the prospective integration and validation of such software within a clinical workflow.^1^ The EpiNav software was integrated as a clinical decision support software to plan SEEG strategies in 13 consecutive patients in whom manual planning was performed independently. The risk scores were computed for the manual and automated implantation plans as a whole and the plan with the lowest overall risk score was selected for surgical implantation. In all 13 cases, the EpiNav automated plans returned the lowest overall risk score and subsequently were implanted using a frameless system without complication. This real-world integration of the software revealed that although the computation times for the automated plans ranged from 34 seconds to 89 seconds, the overall time for trajectory planning, manual review, and modification was 62 minutes ± 17 minutes compared with 221 minutes ± 39 minutes with manual planning alone. In addition, the pipeline highlighted the clinical significance of a seamless export function between the automated planning software and the neuronavigation system for implantation, in this case, the StealthStation (Medtronic, Minneapolis, Minnesota). Manual planning has been replaced by automated planning using the EpiNav software at the authors’ institution.

## Future developments

### Machine Learning of Individual Planning Preferences

A major limitation of computer-assisted planning algorithms is that they are dependent on the quality of the imaging provided and user-defined planning parameters and reflect the planning practices at the institution from which they are developed. This significantly limits the generalizability of the software and its applicability to other SEEG programs. One potential method to overcome this is the use of machine learning to modify the planning parameters and practices to that of the individual user. One such example is the use of spatial priors. The current computer-assisted planning systems are all, to some extent, restricted by the whole-brain parcellation and the subsegmentation of individual structures. If the hippocampus, for example, is not subdivided into head, body, and tail, the user, therefore, cannot choose from these options when specifying the trajectory targets. It is common practice to place SEEG electrodes within both the hippocampal head and body during an investigation of a patient with presumed temporal lobe–onset seizures. Furthermore, it is preferred to place the targets within the medial aspect of the structures to ensure as many contacts are within the target structure when approaching from a lateral neocortical entry zone. The experience gained from previous manual implantations, therefore, can be used to inform the preferred entry and target regions of common trajectories and be used prospectively to guide future implantations. It has been proposed, therefore, that the whole-brain parcellation initially acts as default, and machine learning algorithms then are able to modify the regions based on the surgeons planning preference. Recent preliminary data also have suggested that it may be possible to cluster implantation types into a discrete number set of implantation subtypes.^50^ The implication is that the computer-assisted algorithms also may be able to aid in the refinement of implantation strategies. Both these factors are likely to improve the feasibility and external validity of the planning algorithms in the future.

### Electrode Bending

Given sufficient data, it also has been shown that machine learning techniques can be applied to electrode implantations and accurately predict the degree of electrode bending within the brain. Given that current automated planning systems are based on linear trajectories, knowledge of the potential bending may allow for compensatory corrections to be incorporated at the planning stage.

### Linking Electrophysiologic Data and Resection Volume Planning

The ultimate goal of SEEG is to identify the seizure-onset zone, map the ictal and interictal networks, and identify eloquent functions in order to guide tailored resections. Understanding the exact anatomic location of the electrode contacts within the brain, therefore, is paramount when proposing resective surgery from SEEG data. Future iterations of computer-assisted planning software will integrate SEEG data with the 3-D models and attempt to solve the inverse solutions from the ictal and interictal electrophysiologic patterns. With the integration of structural and functional connectivity data, computer-assisted algorithms then may be able to propose potential resection or ablation volumes.

## Summary

Computer-assisted SEEG trajectory planning has advanced significantly in the past decade due to increased computing power and collaboration between clinicians and engineers. The rise in the number of commercially available robotic trajectory guidance systems will aid in the translation and implementation of computer-assisted planning. Together with robotic devices, the hope is that computer-assisted planning will be integrated into clinical workflows to standardize SEEG trajectories and overcome early learning curves associated with this technique.

## References

[1] Vakharia V.N., Sparks R., Miserocchi A. (2019). Computer-assisted planning for stereoelectroencephalography (SEEG). Neurotherapeutics.

[2] Dale A.M., Fischl B., Sereno M.I. (1999). Cortical surface-based analysis. I. Segmentation and surface reconstruction. Neuroimage.

[3] Fischl B. (2012). FreeSurfer. Neuroimage.

[4] Cardoso M.J., Modat M., Wolz R. (2015). Geodesic information flows: spatially-variant graphs and their application to segmentation and fusion. IEEE Trans Med Imaging.

[5] Gibson E., Li W., Sudre C. (2018). NiftyNet: a deep-learning platform for medical imaging. Comput Methods Programs Biomed.

[6] McClure P., Rho N., Lee J.A. (2019). Knowing what you know in brain segmentation using bayesian deep neural networks. Front Neuroinform.

[7] Mullin J.P., Shriver M., Alomar S. (2016). Is SEEG safe? A systematic review and meta-analysis of stereo-electroencephalography-related complications. Epilepsia.

[8] Zuluaga M.A., Rodionov R., Nowell M. (2015). Stability, structure and scale: improvements in multi-modal vessel extraction for SEEG trajectory planning. Int J Comput Assist Radiol Surg.

[9] Li K., Vakharia V.N., Sparks R. (2019). Stereoelectroencephalography electrode placement: detection of blood vessel conflicts. Epilepsia.

[10] Cardinale F., Cossu M., Castana L. (2013). Stereoelectroencephalography: Surgical methodology, safety, and stereotactic application accuracy in 500 procedures. Neurosurgery.

[11] Isnard J., Taussig D., Bartolomei F. (2018). French guidelines on stereoelectroencephalography (SEEG). Neurophysiol Clin.

[12] Cardinale F., Casaceli G., Raneri F. (2016). Implantation of stereoelectroencephalography electrodes: a systematic review. J Clin Neurophysiol.

[13] Cardinale F., Pero G., Quilici L. (2015). Cerebral angiography for multimodal surgical planning in epilepsy surgery: description of a new three-dimensional technique and literature review. World Neurosurg.

[14] Zuckerman S.L., Bhatia R., Tsujiara C. (2015). Prospective series of two hours supine rest after 4fr sheath-based diagnostic cerebral angiography: Outcomes, productivity and cost. Interv Neuroradiol.

[15] Vakharia V.N., Sparks R., Vos S.B. (2019). The effect of vascular segmentation methods on stereotactic trajectory planning for drug-resistant focal epilepsy: a retrospective cohort study. World Neurosurg X.

[16] Barros G., Lang M.J., Mouchtouris N. (2017). Impact of trajectory planning with susceptibility-weighted imaging for intracranial electrode implantation. Oper Neurosurg.

[17] Wahlin A., Ambarki K., Hauksson J. (2012). Phase contrast MRI quantification of pulsatile volumes of brain arteries, veins, and cerebrospinal fluids compartments: repeatability and physiological interactions. J Magn Reson Imaging.

[18] Guo Z., Leong M.C.W., Su H. (2018). Techniques for stereotactic neurosurgery: beyond the frame, toward the intraoperative magnetic resonance imaging–guided and robot-assisted approaches. World Neurosurg.

[19] De Momi E., Caborni C., Cardinale F. (2013). Automatic trajectory planner for StereoElectroEncephaloGraphy procedures: a retrospective study. IEEE Trans Biomed Eng.

[20] Shamir R.R., Joskowicz L., Tamir I. (2012). Reduced risk trajectory planning in image-guided keyhole neurosurgery. Med Phys.

[21] Trope M., Shamir R.R., Joskowicz L. (2015). The role of automatic computer-aided surgical trajectory planning in improving the expected safety of stereotactic neurosurgery. Int J Comput Assist Radiol Surg.

[22] Essert C., Haegelen C., Lalys F. (2012). Automatic computation of electrode trajectories for Deep Brain Stimulation: A hybrid symbolic and numerical approach. Int J Comput Assist Radiol Surg.

[23] Zombori G, Rodionov R, Nowell M, et al. A computer assisted planning system for the placement of sEEG electrodes in the treatment of epilepsy. In: Stoyanov D, Collins DL, Sakuma I, et al, editors. Information Processing in Computer-Assisted Interventions. Springer International Publishing; 2014. p. 118–127.

[24] Sparks R., Zombori G., Rodionov R. (2017). Automated multiple trajectory planning algorithm for the placement of stereo-electroencephalography (SEEG) electrodes in epilepsy treatment. Int J Comput Assist Radiol Surg.

[25] Sparks R., Vakharia V., Rodionov R. (2017). Anatomy-driven multiple trajectory planning (ADMTP) of intracranial electrodes for epilepsy surgery. Int J Comput Assist Radiol Surg.

[26] Nowell M., Sparks R., Zombori G. (2016). Comparison of computer-assisted planning and manual planning for depth electrode implantations in epilepsy. J Neurosurg.

[27] Scorza D., De Momi E., Plaino L. (2017). Retrospective evaluation and SEEG trajectory analysis for interactive multi-trajectory planner assistant. Int J Comput Assist Radiol Surg.

[28] Elias W.J., Sansur C.A., Frysinger R.C. (2009). Sulcal and ventricular trajectories in stereotactic surgery. J Neurosurg.

[29] Alomar S., Mullin J.P., Smithason S. (2017). Indications, technique, and safety profile of insular stereoelectroencephalography electrode implantation in medically intractable epilepsy. J Neurosurg.

[30] Granados A., Vakharia V., Rodionov R. (2018). Automatic segmentation of stereoelectroencephalography (SEEG) electrodes post-implantation considering bending. Int J Comput Assist Radiol Surg.

[31] Vakharia V.N., Sparks R., O’Keeffe A.G. (2017). Accuracy of intracranial electrode placement for stereoencephalography: A systematic review and meta-analysis. Epilepsia.

[32] Davis D.H., Kelly P.J., Marsh W.R. (1988). Computer-assisted stereotactic biopsy of intracranial lesions in pediatric patients. Pediatr Neurosci.

[33] Giorgi C., Broggi G., Casolino D. (1989). Computer assisted analysis of neuroradiological data in planning neurosurgical procedures. J Neurosurg Sci.

[34] Otsuki T., Jokura H., Takahashi K. (1994). Stereotactic γ-thalamotomy with a computerized brain atlas: technical case report. Neurosurgery.

[35] Nowinski W.L., Yeo T.T., Thirunavuukarasuu A. (1998). Microelectrode-guided functional neurosurgery assisted by electronic clinical brain atlas CD-ROM. Comput Aided Surg.

[36] Nowinski W.L., Yang G.L., Yeo T.T. (2000). Computer-aided stereotactic functional neurosurgery enhanced by the use of the multiple brain atlas database. IEEE Trans Med Imaging.

[37] Vaillant M., Davatzikos C., Taylor R.H., Troccaz J., Grimson E., Mösges R. (1997). A path-planning algorithm for image-guided neurosurgery. CVRMed-MRCAS’97: first joint conference computer vision, virtual reality and robotics in medicine and medical robotics and computer-assisted surgery Grenoble, France, March 19--22, 1997 proceedings.

[38] Guo T., Parrent A.G., Peters T.M. (2007). Automatic target and trajectory identification for deep brain stimulation (DBS) procedures. Med Image Comput Comput Assist Interv.

[39] Beriault S., Al Subaie F., Mok K. (2011). Automatic trajectory planning of DBS neurosurgery from multi-modal MRI datasets. Med Image Comput Comput Assist Interv.

[40] Bériault S., Subaie F Al, Collins D.L. (2012). A multi-modal approach to computer-assisted deep brain stimulation trajectory planning. Int J Comput Assist Radiol Surg.

[41] Liu Y., Konrad P.E., Neimat J.S. (2014). Multisurgeon, multisite validation of a trajectory planning algorithm for deep brain stimulation procedures. IEEE Trans Biomed Eng.

[42] Lüders H.O., Najm I., Nair D. (2006). The epileptogenic zone: general principles. Epileptic Disord.

[43] Perry M.S., Bailey L., Freedman D. (2017). Coregistration of multimodal imaging is associated with favourable two-year seizure outcome after paediatric epilepsy surgery. Epileptic Disord.

[44] Rodionov R., Vollmar C., Nowell M. (2013). Feasibility of multimodal 3D neuroimaging to guide implantation of intracranial EEG electrodes. Epilepsy Res.

[45] Nowell M., Rodionov R., Zombori G. (2015). Utility of 3D multimodality imaging in the implantation of intracranial electrodes in epilepsy. Epilepsia.

[46] Zombori G., Rodionov R., Nowell M., Stoyanov D., Collins D.L., Sakuma I. (2014). A computer assisted planning system for the placement of sEEG electrodes in the treatment of epilepsy. Information processing in computer-assisted Interventions.

[47] Navkar N.V., Tsekos N.V., Stafford J.R., Navab N., Jannin P. (2010). Visualization and planning of neurosurgical interventions with straight access BT - information processing in computer-assisted interventions.

[48] De Momi E., Caborni C., Cardinale F. (2014). Multi-trajectories automatic planner for StereoElectroEncephaloGraphy (SEEG). Int J Comput Assist Radiol Surg.

[49] Vakharia V.N., Sparks R., Rodionov R. (2018). Computer-assisted planning for the insertion of stereoelectroencephalography electrodes for the investigation of drug-resistant focal epilepsy: an external validation study. J Neurosurg.

[50] Scorza D., Amoroso G., Cortes C. (2018). Experience-based SEEG planning: from retrospective data to automated electrode trajectories suggestions. Healthc Technol Lett.

